# Dr. D. Gann Writes

**Published:** 1889-07

**Authors:** 


DR. D. GANN writes us of the successful treatment by himself, in conjunction with Dr. J B Shaw, of a complicated case of urethral stricture with fistulæ, and loss of tissue of the scrotum and perineum. "There were two strictures of the urethra, two fistulæ of the anus, and the entire perineum was diseased to within half inch of anus, extending to middle of scrotum, there being several fistulous openings through which urine escaped during micturition.

